# Effects of early or conventional weaning on beef cow and calf performance in pasture or drylot environments^1^

**DOI:** 10.1093/tas/txac052

**Published:** 2022-04-27

**Authors:** John R Jaeger, Garrett W Preedy, Justin W Waggoner, Keith R Harmoney, K C Olson

**Affiliations:** Western Kansas Agricultural Research and Extension Center – Hays, Kansas State University, Hays, KS, USA; Department of Animal Science and Industry, Kansas State University, Manhattan, KS, USA; Western Kansas Agricultural Research and Extension Center – Hays, Kansas State University, Hays, KS, USA; Western Kansas Agricultural Research and Extension Center – Hays, Kansas State University, Hays, KS, USA; Department of Animal Science and Industry, Kansas State University, Manhattan, KS, USA

**Keywords:** conventional weaning, early weaning, limit-fed

## Abstract

Performance of cows and calves during 63-d early or conventional weaning periods was evaluated. Spring-calving beef cows (*n* = 167) of similar age, body condition score (BCS), and body weight (BW = 599 ± 54.5 kg), and their calves (initial BW = 204 ± 26.7 kg; 153 ± 15 d of age) were assigned randomly to 1 of 4 weaning treatments: weaning at 153 d of age followed by 56 days of limit feeding in confinement (E-D), confinement of cow and calf for a 56-d period of limit feeding followed by weaning at 209 d of age (C-D), weaning at 153 d of age followed by a 56-d grazing period (E-P), and a 56-d grazing period for both cow and calf followed by weaning at 209 d of age (C-P). Cows and calves assigned to pasture treatments grazed native range pastures without supplement. Cows and calves assigned to drylot treatments were fed complete diets. Calves assigned to E-D were fed a concentrate-based diet at 2.5% of BW, whereas cows assigned to E-D were fed a forage-based diet at 1.6% of BW. Cows assigned to C-D were offered the diet fed to E-D cows at 2.0% of BW. Calf average daily gain (ADG) was influenced by diet and weaning treatments (diet × weaning, *P* ≤ 0.03). Cows and calves assigned to all treatments were limit fed common diets for 7 d at the end of our study to equalize gut fill. In general, calves managed in confinement and fed concentrate-based diets (i.e., E-D and C-D) had greater ADG than non-supplemented calves maintained on pasture (i.e., E-P and C-P). Cow BW and BCS change (days 0 to 63) were influenced by both diet and weaning status (*P* ≤ 0.05). Non-lactating cows maintained on pasture had lesser BW loss than other treatments, whereas non-lactating cows fed in confinement had lesser BCS on day 63 and greater BCS loss from days 0 to 63 than other treatments. Conversely, rump-fat depth on day 63 was greater (*P* < 0.01) for non-lactating cows maintained on pasture than for lactating cows in either pasture or drylot environments. Similarly, change in rump-fat depth was greatest (diet × weaning, *P* < 0.01) for non-lactating cows on pasture and least for lactating cows in either pasture or drylot environments. Results were interpreted to indicate that early-weaning spared cow BW and rump fat compared to weaning at conventional ages. Performance of cows appeared to be similar when limit-fed under drylot conditions or maintained in a pasture environment. Conversely, calf performance was generally greater in confinement than on pasture.

## INTRODUCTION

Widespread drought occurs frequently across the Midwest, as a result pasture availability and productivity can be reduced. This, coupled with increasing land prices and lease rates, has prompted the evaluation of alternative management strategies that decrease grazing pressure on perennial pastures or that reduce feed and pasture costs. Weaning early and moving cows from pasture to a drylot environment is used commonly for reducing grazing pressure on perennial pastures. A premature end to lactation reduces cow nutrient requirements and reduces grazing pressure. Removal of the calf further reduces grazing pressure, as calves are significant consumers of forage dry matter (DM) during mid and late lactation (Boggs et al., 1980). The combination can be used to extend grazing by 0.4 d for each day weaning is executed earlier than normal (Rasby, 2007). Early weaning may result in calves having less value at weaning compared to calves weaned at conventional ages (Story et al., 2000). Retaining ownership of young calves through backgrounding can be useful for increasing their value.

Limit feeding non-lactating cows or cow-calf pairs in confinement can also reduce grazing pressure on pastures, while maintaining cow body condition score (BCS) or body weight (BW) (Loerch, 1996; Tjardes et al. 1998). Brethour et al. (1990) reported similar BW gains and greater pregnancy rates for non-lactating cows fed in confinement compared with lactating cows grazing native pastures. Limit-feeding non-lactating cows at 1.9% BW achieved acceptable gains in BW, BCS, and rump fat (Waggoner and Jaeger, 2014). Therefore, the objective of our study was to evaluate the performance of beef cows and calves subject to a 56-d early or conventional weaning period in either pasture or drylot environments.

## MATERIALS AND METHODS

The Kansas State University Institutional Animal Care and Use Committee reviewed and approved all animal handling and animal care practices used in our experiment. All animal procedures were conducted in accordance with the Guide for the Care and Use of Animals in Agricultural Research and Teaching (FASS, 2020). Animal care practices used in our experiment were approved by the Kansas State University Animal Care and Use Committee (protocol no. 2978.1). The experiment was conducted at the Western Kansas Agricultural Research and Extension Center, Hays, Kansas.

### Animals

Spring-calving Angus-cross cows (*n* = 167) with an initial body weight (BW) of 599 ± 54.5 kg; an average age of 5 ± 2.4 years, and an initial body condition score (BCS) of 5.5 ± 0.54 and their calves (*n* = 167; initial BW = 204 ± 26.7 kg; 153 ± 15 d of age) originating from the commercial cow-calf herd of the Western Kansas Agricultural Research Center in Hays, KS were used in this experiment. Bull calves were castrated within 24 h of birth and all calves were vaccinated against clostridial diseases (Ultrabac 7; Pfizer Animal Health, Exton, PA) at approximately 60 d of age. At the initiation of the study on August 19, cow-calf pairs were stratified by calf age, cow BW, and cow BCS and assigned randomly to 1 of 4 weaning treatments with 4 pens or pasture replicates per treatment. Treatments were as follows: weaning at 153 d of age followed by 56 d of limit feeding in confinement for both cow and calf (E-D), confinement of cow and calf together for a 56-d period of limit feeding followed by weaning at 209 d of age (C-D), weaning at 153 d of age followed by a 56-d grazing period for both the separated cow and calf (E-P), and a 56-d grazing period for cow and calf together followed by weaning at 209 d of age (C-P).

Cows and calves across all treatments were weighed individually and calves were given initial vaccinations against respiratory pathogens (Bovi-Shield Gold 5; Pfizer Animal Health, Exton, PA) and clostridial pathogens (Ultrabac 7; Pfizer Animal Health, Exton, PA). Calves were treated for internal and external parasites (Dectomax Injectable; Zoetis Inc., Kalamazoo, MI), given injectable trace minerals (Multimin 90; Multimin USA Inc., Fort Collins, CO), and steers were given a growth-promoting implant (Ralgro; Intervet Inc., Merck Animal Health, Summit, NJ). Calves were re-vaccinated for viral respiratory pathogens and clostridial pathogens 14 days after study initiation.

### Drylot Treatments

Cows and calves assigned to E-D and C-D were placed into the feedlot at the Western Kansas Agricultural Research Center for 56 d. Calves assigned to E-D were separated from their dams and placed in feedlot pens (*n* = 4, minimum area = 200 m^2^/calf; bunk space = 0.46 m/calf) and afforded ad libitum access to water. Calves were fed a weaning diet (Table 1) formulated to promote a 1-kg average daily gain (ADG) at a dry matter intake (DMI) of 2.5% of BW. Bunks were evaluated each morning at 0630 h, and feed was delivered once daily at 0700 h. Bunks were managed using a slick-bunk management method to minimize feed refusals (Pritchard and Bruns, 2003). If all feed delivered to a pen was consumed, delivery at the next feeding was increased to approximately 102% of the previous delivery. Diet samples were collected from bunks weekly and frozen at −20 °C. Samples were composited by weight at the conclusion of the study and submitted to a commercial laboratory (SDK Laboratories, Hutchinson, KS) for analysis (Table 1). Diet net energy (NE) values were calculated from detergent fiber analyses using equations provided by NASEM (2016).

**Table 1.** Composition of diet fed to early-weaned calves in confinement

Ingredient composition	% DM
Sorghum silage	21.9
Dry rolled sorghum grain	63.4
Wet distillers grains	6.1
Soybean meal	5.1
Supplement^*^	3.4
Nutrient composition	DM basis
CP, % DM	18.1
NE_m_^†^, Mcal/kg DM	1.81
NE_g_^†^, Mcal/kg DM	1.09

Supplement contained ammonium sulfate, limestone, urea, salt, Rumensin 90 (300 mg head^−1^∙ d^−1^), Tylan 40 (90 mg head^−1^∙d^−1^), and a trace-mineral premix.

Net energy of maintenance (NE_m_) and net energy of gain (NE_g_) were calculated using equations suggested by NASEM (2016).

Cows assigned to E-D were separated from their calves and placed in earth floor feedlot pens (*n* = 4, minimum area = 1033 m^2^/cow; linear bunk space = 0.65 m/cow) and afforded ad libitum access to water. Cows were limit fed a roughage-based diet at 1.6% of initial BW that was formulated to meet nutrient requirements of pregnant cows in late lactation (NASEM, 2016; Table 2). Feed was delivered once daily at 0700 h. Diet samples were collected from bunks weekly and frozen at −20 °C. Diet samples were composited by weight at the conclusion of the study and submitted to a commercial laboratory (SDK Laboratories, Hutchinson, KS) for analysis (Table 2). Diet NE values were calculated from detergent fiber analyses using equations provided by NASEM (2016).

**Table 2.** Composition of the diet fed to beef cows in confinement

Ingredient composition	% DM
Ground hay^*^	80.6
Dry rolled sorghum grain	10.4
Wet distillers grains	7.9
Calcium carbonate	0.30
Salt	0.30
Vitamin and mineral premix	0.30
Nutrient composition	DM basis
CP, % DM	13.2
NE_m_^†^, Mcal/kg DM	1.68

Native prairie hay blended with forage sorghum hay.

Net energy of maintenance (NE_m_) was calculated using equations suggested by NASEM (2016).

Cows and calves assigned to C-D were placed as pairs into feedlot pens (*n* = 4, minimum area = 1033 m^2^/cow; bunk space = 0.65 m/cow) and afforded ad libitum access to water. Cows were limit fed a forage-based diet at 2.0% of initial BW that was formulated to meet nutrient requirements of pregnant cows in late lactation (NASEM, 2016). Calves were offered the same diet fed to E-D (Table 1) at a daily DM allowance of 2.0% of initial BW. Creep panels were used to allow calves undisturbed access to the weaning diet. Cow and calf bunks were evaluated each morning at 0630 h, and feed was delivered once daily at 0700 h. Diet samples were collected from bunks weekly and frozen at −20 °C. Samples were composited by weight and nutrient composition was analyzed as described above.

### Pasture Treatments

Cows and calves assigned to E-P and C-P were placed onto the native pastures at the Western Kansas Agricultural Research Center for 56 d. The native vegetation was composed primarily of sideoats grama, western wheatgrass, blue grama, Japanese brome, and buffalograss. Calves assigned to E-P were separated from their dams and placed in feedlot pens for 4 d (*n* = 4, minimum area = 200 m^2^/calf; bunk space = 0.46 m/calf) and afforded ad libitum access to water. Calves were fed native prairie hay ad libitum. Hay was delivered once daily at 0700 h. On the afternoon of day 4, calves were released into 1 of 4 assigned pastures. Each pasture (11 ± 0.4 ha) provided continual access to water and was stocked at 0.8 ha per calf for 52 d.

Two permanent 100-m transects were established in each pasture at the onset of the study in order to estimate forage quality and above-ground forage biomass. Pasture forage quality and biomass were estimated by clipping all plant material from within randomly placed sampling frames (0.25 m^2^; *n* = 10 per pasture) at a height of 1 cm on 19 August, 16 September, and 14 October. Range forage samples dried in a forced-air oven (50 °C; 96 h) and weighed to estimate biomass availability (Table 3). Samples were subsequently composited by sampling date on an equal-weight basis at the conclusion of the experiment and submitted to a commercial laboratory (SDK Laboratories, Hutchinson, KS) for analysis of DM, crude protein (CP), neutral detergent fiber (NDF), and acid detergent fiber (ADF; Table 3).

**Table 3.** Forage biomass (kg forage DM/100 kg BW) and nutrient composition available to weaned calves, non-lactating cows, and cow-calf pairs during a 56-d grazing period

Item	Weaned calves^*^	Non-lactating cows^†^	Cow-calf pairs^‡^	SEM
Forage biomass				
19 August, kg	812.3^a^	443.3^b^	356.2^b^	65.65
16 September, kg	806.5^a^	389.8^b^	317.9^b^	54.04
14 October, kg	661.1^a^	345.2^b^	345.2^b^	49.70
Nutrient composition				
CP, % DM				
19 August	6.8	6.2	5.8	
16 September	5.9	5.5	5.2	
14 October	5.5	4.6	5.4	
NDF, % DM				
19 August	71.1	71.6	70.4	
16 September	76.2	76.7	74.9	
14 October	74.9	77.2	75.1	
ADF, % DM				
19 August	46.2	45.8	44.6	
16 September	51.2	51.1	49.3	
14 October	51.6	52.4	50.5	

Calves were early weaned in a pasture environment and not supplemented for 56 d.

Dams of early-weaned calves in a pasture environment and not supplemented for 56 d.

Cow-calf pairs grazed together in a pasture environment and not supplemented for 56 d.

Within a row, means without a common superscript differ (*P* ≤ 0.01).

Cows assigned to E-P were separated from their calves and placed in feedlot pens for 4 d (*n* = 4, minimum area = 1033 m^2^/cow; bunk space = 0.65 m/cow) and afforded ad libitum access to water. Cows were fed the same native prairie hay offered to E-P calves for ad libitum intake during this period. Hay was delivered once daily at 0700 h. Cows were released into assigned pastures, on the afternoon of day 4 and remained there 52 d. Each pasture (*n* = 4, 15 ± 0.4 ha) was stocked at 1.2 ha/cow and provided continual access to water. Total forage biomass (Table 3) and pasture forage quality (Table 3) were collected as described above on 19 August, 16 September, and 14 October.

Cows and calves assigned to C-P were placed as pairs directly onto native range pasture (*n* = 4, 15 ± 0.4 ha) for 56 d. Pastures were stocked at 1.6 ha per pair and provided continual access to water. Total forage biomass (Table 3) and pasture forage quality (Table 3) were collected as described above on 8/19, 9/16, and 10/14.

### Final Phase

Following the 56-d study period, cows and calves were individually weighed. Animals assigned to E-P and C-P were transported to the Western Kansas Agricultural Research Center feedlot. Cows and calves assigned to C-P and C-D were separated at this time and assigned to a new pen (*n* = 4 per treatment for cows, 4 per treatment for calves). To attempt to equalize gut-fill between treatments, all calves were fed a common diet (Table 1) at 2.0% of BW of their day-56 BW and all cows were fed a common diet (Table 2) at 1.6% of BW of their day-56 BW for 7 d.

### Data Collection

Calf BW were individually measured on days 0, 28, 56, and 63. Cows were weighed individually on days 0 and 63. Cows and calves were weighed at 0600 h prior to feed delivery. Cow BCS were assigned by two trained observers using a 9-point scale (1 = emaciated, 9 = obese; Wagner et al., 1988) on days 0 and 63. Also on days 0 and 63, rump fat thickness at the midpoint between the tuber coxae (hip bone) and the tuber ischia (pin bone) was measured ultrasonically using an Aloka 500V (Aloka Co., Ltd., Wllingford, CT) B-mode instrument equipped with a 3.5-MHz general purpose transducer array (UST 5021-12mm window). Cattle Performance Enhancement Company (CPEC, Oakley, KS) software was used to collect ultrasound images. Rump fat thickness was estimated with procedures that incorporated image analysis software integral to the CPEC software (Brethour, 1994).

### Statistical Analysis

Cow and calf performance were analyzed as a mixed model with a 1-way treatment structure in factorial arrangement of a completely randomized design (PROC MIXED; SAS Inst. Inc., Cary, NC). Pen or pasture was the experimental unit. Class factors included pen or pasture, weaning treatment, and weaning diet. The model statement included terms for the fixed effects of weaning treatment, weaning diet, and weaning treatment × weaning diet.

Native range biomass data were analyzed as a mixed model with a 1-way treatment structure in a completely randomized design (PROC MIXED; SAS Inst. Inc., Cary, NC). Pasture was the experimental unit. Class factors included treatment and pasture. The model statement included a term for the fixed effect of treatment only.

When protected by a significant *F*-test (*P* < 0.05), least squares treatment means were separated using the method of least significant difference. Means were considered different when *P* ≤ 0.05.

## RESULTS AND DISCUSSION

### Forage Biomass

Available native range forage biomass was greater (*P* ≤ 0.01) for E-P calves than for either E-P cows or C-P cow-calf pairs for the duration of our study (Table 3). This was expected because of less grazing pressure afforded by calves compared with either cows or cow-calf pairs. Available forage biomass was similar (*P* ≥ 0.21) between pastures for C-P cow-calf pairs and E-P cows throughout our experiment. Range forage biomass declined in quantity throughout the experiment in all treatments.

### Calf Performance

Calf BW was not different (*P* ≥ 0.06) between treatments at the beginning of the study or on day 28 (Table 4). By day 63, an interaction (*P* = 0.05) occurred between diet and weaning treatment. Calves managed in confinement, both weaned and non-weaned, had greater BW than calves managed on pasture. This observation is similar to Bailey et al. (2016) who reported that calves weaned in a pasture environment for 28 d before being moved to a feedlot had lower body weight gain during the weaning and receiving period than did their drylot-weaned contemporaries. Calves suckling their dams had greater BW than weaned, non-supplemented calves grazing native pastures. Average daily gains were influenced also by diet and weaning treatments (diet × weaning; *P* ≤ 0.03). In general, calves managed in confinement and fed concentrate-based diets (i.e., E-D and C-D) had greater ADG than non-supplemented calves maintained on pasture (i.e., E-P and C-P). Weaned calves on pasture had 50.0% and 62.5% lower (*P* < 0.01) ADG than suckling calves on pasture from days 0 to 28 and from days 0 to 63, respectively (Table 4). Mathis et al. (2008) also reported that calves preconditioned on native range weighed less than calves preconditioned in drylot at the end of a 45-d preconditioning period. However, early weaning can conserve a significant amount of pasture forage for the weaned cow. Boggs et al. (1980) reported that calves born in mid-March to early April were consuming 1.5% of BW by 3 mo of age and increased to 2.2% of BW by 6 mo of age. Rasby (2007) also found that early-weaning calves reduces rangeland stocking rates 20%–30%. Using these figures, essentially every 4 d a calf is weaned conserves 1 grazing day for a 635 kg dry cow or for each 30 d a calf is weaned early, enough forage is conserved to extend mature cow grazing by 1 wk. This can have a significant effect on ability to retain productive cows during drought.

**Table 4.** Performance of beef calves that were weaned early or paired with dams in either confinement or pasture environments

						P-value		
Item	Weaned calves—confined*	Non-weaned calves—confined^†^	Weaned calves—pasture^‡^	Non-weaned calves—pasture^§^	SEM	Diet	Weaning	Diet x Weaning
Initial BW, kg	208	205	207	204	4.1	0.83	0.50	0.99
day 28 BW, kg	242	244	227	243	4.6	0.07	0.06	0.16
day 63 BW, kg	277^c^	285^c^	226^a^	254^b^	5.0	< 0.01	< 0.01	0.05
ADG days 0 to 28, kg	1.2^c^	1.4^b^	0.7^a^	1.4^b^	0.05	< 0.01	< 0.01	< 0.01
ADG days 28 to 63, kg	1.0^b^	1.2^c^	−0.3^a^	0.3^a^	0.04	< 0.01	< 0.01	0.03
ADG days 0 to 63, kg	1.1^c^	1.3^d^	0.3^a^	0.8^b^	0.04	< 0.01	< 0.01	< 0.01

Calves were weaned in a drylot environment and fed a growing diet for 56 d.

Cow-calf pairs confined together in a drylot environment and fed complete diets for 56 d.

Calves were weaned in a pasture environment and not supplemented for 56 d.

Cow-calf pairs grazed together in a pasture environment and were not supplemented for 56 d.

Within a row, means without a common superscript differ (*P* ≤ 0.01).

### Cow Performance

Cow BW, BCS, and rump-fat thickness were not different (*P* ≥ 0.36) between treatments at the beginning of the study (Table 5). Cow BW on day 63 was greatest (*P* < 0.01) for non-lactating cows on pasture, intermediate for non-lactating cows in confinement, and least for cows that continued to suckle calves. Overall, BW change was influenced by both diet and weaning status (diet × weaning; *P* = 0.05). Non-lactating cows maintained on pasture had less BW loss than other treatments (−1.0 vs. ≥ 30.0 kg, respectively); BW loss by confined non-lactating cows and lactating cows maintained on pasture was less than confined lactating cows. Previous researchers reported that limit-feeding non-lactating cows at 1.9% BW achieved acceptable gains in BW, BCS, and rump fat (Waggoner and Jaeger, 2014). However, these researchers did not examine the performance of limit-fed lactating cows fed in confinement. Brethour et al. (1990) also reported similar BW gains and greater pregnancy rates for non-lactating cows fed in confinement compared with lactating cows grazing native pastures. Reduced body weight loss by non-lactating cows is primarily due to the reduction in maintenance energy requirements. It has been estimated that lactating Hereford cows have a 30% increase in maintenance energy requirements compared to nonlactating Hereford cows (Neville and McCullough, 1969; Neville, 1974).

**Table 5.** Performance of pregnant beef cows in confinement and pasture environments either post-weaning or while suckling calves

Item	Post-weaning—confined^*^	Suckling—confined^†^	Post-weaning—pasture^‡^	Suckling—pasture^§^	SEM	P		
						Diet	Weaning	Diet x Weaning
BW, kg								
day 0	613	603	597	603	8.6	0.37	0.85	0.36
day 63	583^b^	555^a^	596^c^	570^ab^	8.5	< 0.01	< 0.01	0.93
Change, days 0 to 63	−30.0^b^	−48.4^c^	−1.0^a^	−33.7^b^	3.58	< 0.01	< 0.01	0.05
BCS								
day 0	5.5	5.4	5.5	5.5	0.08	0.56	0.78	0.47
day 63	4.5^a^	5.0^b^	5.1^b^	5.0^b^	0.07	< 0.01	< 0.01	< 0.01
Change, days 0 to 63	−1.0^a^	−0.4^b^	−0.4^b^	−0.6^b^	0.70	< 0.01	< 0.01	< 0.01
Rump fat depth, mm								
day 0	5.43	5.67	4.91	5.44	0.054	0.49	0.48	0.78
day 63	6.69^ab^	6.05^a^	8.33^b^	5.89^a^	0.057	0.19	< 0.01	0.12
Change, days 0 to 63	1.262^b^	0.393^c^	3.411^a^	0.449^c^	0.030	< 0.01	< 0.01	< 0.01

Cows were maintained in a drylot environment and fed a forage-based diet for 56 d.

Cow-calf pairs confined together in a drylot environment and fed complete diets for 56 d.

Cows were maintained in a pasture environment and not supplemented for 56 d.

Cow-calf pairs grazed together in a pasture environment and were not supplemented for 56 days.

Within a row, means without a common superscript differ (*P* ≤ 0.01).

Cow BCS on day 63 and BCS change from days 0 to 63 were influenced (*P* < 0.01) by diet and weaning status. Non-lactating cows fed in confinement had lower BCS on day 63 and greater BCS loss from days 0 to 63 than all other treatments. In contrast, other authors have reported that limit-feeding non-lactating cows or cow-calf pairs in confinement sustained cow body weight and body condition score (Loerch, 1996; Tjardes et al. 1998). In fact, Loerch (1996) reported limit-fed primarily corn and consuming 1.2% of their BW in confinement displayed less BW loss, gave birth to heavier calves, weaned heavier calves and a numerically greater conception rate compared to dry, pregnant cows full-fed primarily hay and consuming 2.3% dry matter of their BW in confinement. During drought forages, both standing and harvested, become limited and expensive. Early weaning calves to reduce nutritional requirements for lactation can reduce total forage needed. A lactating 635 kg cow requires 13.7 kg dry forage per day while a non-lactating 635 kg cow only requires 12.6 kg dry forage per day (NASEM, 2016). Additionally, shifting to an energy-dense limit-fed primarily concentrate diet fed in confinement can further reduce reliance on forages.

Trends in BW and BCS may be interpreted to indicate that DMI for the cows assigned to E-C treatment were not adequate to maintain BW or BCS. Conversely, rump-fat data do not support this conclusion. Rump-fat depth on day 63 was greater (*P* < 0.01) for non-lactating cows maintained on pasture than for lactating cows in either pasture or drylot environments; non-lactating cows in confinement were intermediate to and not different from these treatments. Similarly, change in rump-fat depth was greatest (diet × weaning; *P* < 0.01) for non-lactating cows on pasture and least for lactating cows in either pasture or drylot environments. Non-lactating cows maintained in confinement were intermediate to and different from these treatments (Table 5).

## CONCLUSIONS

Results were interpreted to indicate that early-weaning spared cow BW and rump fat compared to weaning at conventional ages. Performance of cows was acceptable when either limit-fed under drylot conditions or maintained in a pasture environment. Conversely, calf performance was generally greater in confinement than on pasture.
